# Molecular Pharmacology of Vitamin C and Relevance to Health and Obesity—A Narrative Review

**DOI:** 10.3390/ijms25147523

**Published:** 2024-07-09

**Authors:** Robert Beaumont Wilson, Yicong Liang, Devesh Kaushal, Anitra Carr

**Affiliations:** 1 School of Clinical Medicine, University of New South Wales (Sydney), Elizabeth St, Liverpool, NSW 2170, Australia; 2 Bankstown Hospital, University of New South Wales (Sydney), Bankstown, NSW 2200, Australia; z5083614@zmail.unsw.edu.au; 3 Campbelltown Hospital, Western Sydney University, Sydney, NSW 2560, Australia; d.kaushal@westernsydney.edu.au; 4 Nutrition in Medicine Research Group, Department of Pathology and Biomedical Science, University of Otago, Christchurch 8140, New Zealand; anitra.carr@otago.ac.nz

**Keywords:** ascorbate, auxotrophs, GLUT, lipid peroxidation, obesity, oxidative stress, SVCT, vitamin C, Western diet

## Abstract

The role of food constituents as pharmacological agents is an important consideration in health and obesity. Vitamin C acts as a small molecule antioxidant but is also a co-factor for numerous transition metal-dependent enzymes involved in healthy weight and energy metabolism. Vitamin C cannot be manufactured by humans and is mainly obtained from the dietary intake of fresh fruit and vegetables. There is great variability between different nutritional guidelines in the recommended daily allowance of vitamin C. Vitamin C deficiency results from an inadequate intake of vitamin C-containing foods and also increased utilization by oxidative and carbonyl stress. Risk factors for vitamin C deficiency include cigarette smoking, malnutrition, obesity, type 2 diabetes mellitus, age, race, sex, social isolation, major surgery, and Western-type diets. Despite the common belief that vitamin C deficiency is rare in affluent countries, surveys of large populations and specific patient groups suggest otherwise. Patients with obesity typically consume highly processed, energy-dense foods which contain inadequate micronutrients. As obesity increases, larger amounts of oral vitamin C are required to achieve adequate plasma and tissue concentrations, as compared to persons with a healthy weight. This is important in the control of oxidative stress and the maintenance of homeostasis and organ function. In this narrative review, the dosage, absorption, distribution, excretion, and catabolism of vitamin C are reviewed, together with the latest findings on vitamin C pharmacology in patients with obesity.

## 1. Introduction

Obesity is defined as an ‘abnormal and excessive fat accumulation that may impair health’. In Caucasians, a body mass index (BMI) ≥ 25 kg/m^2^ is defined as overweight, while obesity is defined as a BMI ≥ 30 kg/m^2^ [1]. Since 1990, the worldwide prevalence of obesity in adults has more than doubled and was 16% in 2022 [1]. This emerging pandemic of obesity is related to progressive urbanization and sedentary lifestyles, as well as the overconsumption of ultra-processed, energy-dense, but micronutrient-poor foods [1,2,3]. Such combined high-fat and high-sucrose foods rarely occur in nature but are typical of Western diets. They stimulate separate vagally mediated gut–brain food reward pathways, which promote the subconscious drive to overeat obesogenic diets [4].

Obesity is closely associated with metabolic syndrome, insulin resistance, hypertension, hyperlipidemia, central adiposity, non-alcoholic fatty liver disease (NAFLD), obstructive sleep apnoea (OSA), accelerated atherosclerosis, type 2 diabetes mellitus (T2DM), epigenetic aging, and premature death [1,2,3]. In the 2003 USA National Health and Nutrition Examination Survey (NHANES) involving data from 1988 to 1994, the prevalence of metabolic syndrome was 5%, 23%, and 60% in men who are normal weight, overweight, and obese in the US population [5]. There are disparities between the various national and international guidelines for adequate vitamin C intake, which is made more complex by the additional requirements in obesity and metabolic syndrome. This narrative review aimed to examine the pharmacology of vitamin C, the scientific basis for dietary vitamin C intake recommendations, and the role of vitamin C supplementation in obesity and metabolic syndrome [5,6,7,8,9,10,11,12,13,14,15].

## 2. Vitamin C Physiology

**Figure 1:**
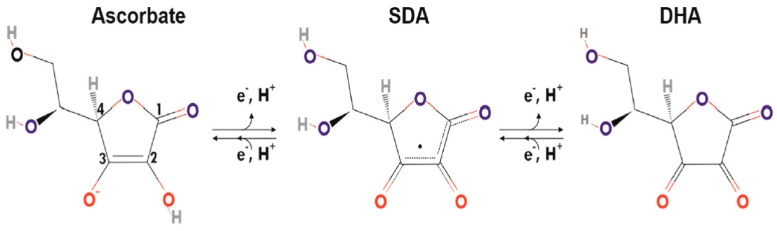
The three vitamin C redox states. At a physiological pH, vitamin C exists as an ascorbate anion in its fully reduced form. Donation of an electron yields semidehydroascorbate (SDA•), which represents the mono-oxidized radical form; donation of another electron generates dehydroascorbate (DHA), the fully oxidized form. Reproduced from Kietzmann et al. (2023) with permission [10].

At a physiological pH, ascorbic acid (AscH_2_) dissociates to its resonance-stabilized anion ascorbate (AscH^−^) [10]. Ascorbate is a strong reducing agent as it is able to donate electrons to other molecules and acts as a non-enzymatic antioxidant or as a co-factor for transition metal-dependent enzymes. It maintains redox potential by shuttling electrons or protons between its ascorbyl radical (semidehydroascorbate (SDA•)) and dehydroascorbic acid (DHA) (Figure 1) [10].

The average plasma vitamin C concentration in a healthy human adult is between 40 and 65 µmol/L [12,16,17]. A plasma vitamin C concentration of 50 µmol/L corresponds to a weight-related (22 mg/kg) total body pool of 1200–2000 mg, with a physiological catabolic rate of 2.9 ± 0.6% per day in healthy, non-smoking male adults [12]. In the absence of repletion by dietary consumption, vitamin C deficiency can develop within 30–45 days. Scurvy can manifest when plasma ascorbate concentrations fall below 11 µmol/L, corresponding to a total body pool of less than 300 mg. Plasma vitamin C concentrations are typically higher in adult females compared to males, related to smaller body mass and lower volumes of distribution at a vitamin C oral intake below saturation (30–100 mg/day), as well as healthier dietary habits and lower rates of smoking in females [7]. The mean (±1 SD) plasma vitamin C renal thresholds (above which vitamin C appears in the urine) in healthy men and women are 49 ± 5 µmol/L and 58 ± 8 µmol/L, respectively [16]. In healthy young adults, steady-state vitamin C plasma concentrations of 60–80 µmol/L are typically achieved with an oral intake of vitamin C between 200 and 400 mg/day [12,16,17].

## 3. Vitamin C Intake and Transport

**Figure 2:**
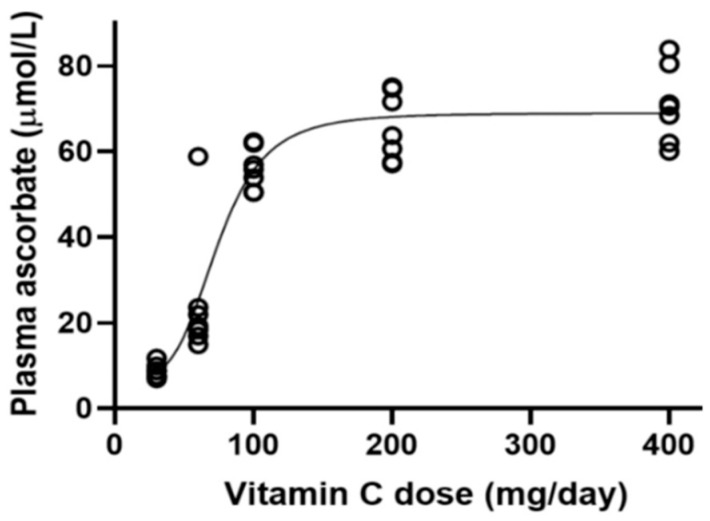
Steady-state plasma ascorbate concentrations in volunteers as a function of daily vitamin C dose. Data was obtained from Levine (1996) [26] and fitted to the curve. Reproduced from Carr et al. (2022) with permission [20,22].

In 2000, the Food and Nutrition Board of the U.S. National Academy of Sciences advised that adult males and females should have a recommended dietary allowance (RDA) of vitamin C of 90 mg and 75 mg/day, respectively, with an increased vitamin C RDA in pregnancy (85 mg/day), lactation (115 mg/day), and an extra requirement in smokers (+35 mg/day) [18]. The vitamin C tolerable upper intake limit was set at 2000 mg/day, based on potential osmotic diarrhea or gastrointestinal disturbance above 3000 mg/day in some people [18]. The previous US and Canadian adult vitamin C RDA of 60 mg/day set in 1989 was increased. This was because it was recognized that although 60 mg/day of oral vitamin C was sufficient to prevent scurvy, in some people, 60 mg/day was below the renal threshold. This dose did not produce sufficient leucocyte saturation levels for cellular protection against oxidant damage from superoxides generated by the leucocyte nicotine adenine dinucleotide phosphate (NADPH) oxidase 2 (NOX-2) respiratory burst during phagocytosis. The vitamin C RDA of 75 mg/day for women was derived from an extrapolation of the estimated average requirement (EAR) in men on the basis of sex differences in body weight [19]. However, it was subsequently suggested that the assumed standard deviation of 10% on the EAR used to calculate the vitamin C RDA in men was inadequate. Based on vitamin C depletion–repletion studies, the actual EAR SD should be 19.4%, resulting in an RDA in men of (EAR (75 mg) + 2SD (38.8%) = RDA (75 mg + 29 mg = 104 mg) [20,21]. In young women, the US vitamin C RDA of 75 mg lays on the steep part of the sigmoid curve of tissue saturation, and it was only with oral doses above 100 mg/day that the asymptote was achieved and a plasma concentration was obtained (60–70 µmol/L), at which the tissue sodium-dependent vitamin C co-transporter 2 (SVCT2) should function at or near *V*max (Figure 2) [21,22].

In 2013, the European Food Safety Authority (EFSA) [16] and in 2015, the Nutrition Societies of Germany, Austria, and Switzerland (DACH) increased their vitamin C RDA to 110 mg/day for men and 95 mg/day for women [23,24,25,26]. The Australian NHMRC has a suggested dietary target (SDT) of vitamin C obtained from whole foods of 220 mg/day for men and 190 mg/day for women. This was in recognition of the need to not only prevent vitamin C deficiency but also maintain adequate plasma concentrations of vitamin C to reduce the risk of non-communicable or chronic diseases. However, the Australasian vitamin C RDA remains at 45 mg/day for both men and women. This is based on an assumed vitamin C body pool in a healthy 70 kg man of 900 mg, which is halfway between the point at which clinical signs of scurvy appear (300 mg) and full tissue saturation (1500 mg), with a vitamin C EAR of 30 mg/day, an oral absorption rate of 85%, and a catabolic rate of 2.9% [16,23,24,25,26]. Maintaining a body pool of 900 mg was estimated to provide protection from scurvy for 30 days in men, even with zero future intake of vitamin C. Such calculations were based on the earlier Sheffield (1948) and Iowa (1969) studies of experimental vitamin C deficiency in humans, as well as short-term ^14^C-labelled ascorbate studies. These studies were performed before sodium-dependent vitamin C transport and the intracellular compartmentalization of ascorbate was known [3,23]. Indeed, more recent studies have indicated the total body pool of vitamin C to be between 3700 and 5000 mg [3,23].

Vegetables and fruits are the major sources of vitamin C, and dietary recommendations of five standard servings of fruit and vegetables per day provide approximately 200 mg of vitamin C [13]. In the USA National Health and Nutrition Examination Survey (NHANES) 2017–2018 data, this dietary threshold was reached in only 8% of the surveyed US adult population, corresponding to a mean serum vitamin C of 66 μmol/L (95% CI, 62, 69). In comparison, the overall cohort mean serum vitamin C was 51 μmol/L (95% CI, 48, 54) [13]. The vitamin C content of fruit and vegetables is quite variable and is dependent on the food type and ripeness, latitude, season, time of harvest, transport, storage, and method of preparation [10,19]. In the USA, the most commonly consumed vegetables and fruit include French fried potatoes, tomatoes, onions, lettuce, apples, bananas, and grapes, which contain relatively low levels of vitamin C [27]. In contrast, whole fruits such as citrus, guava, papaya, kiwifruit, strawberries, and pineapple, and vegetables including capsicums, broccoli, cauliflower, and Brussels sprouts have higher content of vitamin C but are less frequently consumed [20,28]. The bioavailability of vitamin C from whole foods is estimated to be 76%, as compared to 70–90% for synthetic vitamin C supplements [29].

Because the ascorbate anion is a water-soluble, polar molecule and cannot cross lipid bilayers by simple diffusion, its absorption, distribution, and re-uptake are controlled by carrier-mediated transport. The ability to saturate tissues with vitamin C is related to the dissociation of ascorbic acid to ascorbate anion in the stomach, small intestinal active transport of ascorbate by SVCT1, facilitated diffusion of DHA from the intestines into blood by glucose transporter protein type 2 (GLUT-2) and GLUT-8, and from blood to tissues by GLUT-1, GLUT-3, and GLUT-4, renal tubular reabsorption of filtered ascorbate by SVCT1, and active transport by SVCT2 into tissues against a large concentration gradient [10,30,31,32]. Rapid intracellular conversion of DHA to ascorbate enhances GLUT-facilitated transmembrane diffusion of DHA. Ascorbate levels can be depleted by oxidation or renal, intestinal, or sweat losses. Intestinal transport of ascorbate by SVCT1 is also dose-related and saturable, meaning that absorption falls to 50% or less with increasing oral doses of vitamin C above 1 g/day. This may contribute to osmotic diarrhea in some individuals with replete vitamin C levels taking large oral doses (≥4 g/day) of vitamin C [22]. The activity of SVCT-1 and SVCT-2 transporters is also subject to *substrate regulation*. For example, high intracellular ascorbate concentrations may downregulate SVCT-1 transporter translation, resulting in increased intestinal or renal excretion of unmetabolized ascorbate [33].

Tissues that utilize large amounts of vitamin C or are subject to oxidative stress normally have intracellular concentrations of vitamin C that are 10–100 times higher than plasma, including brain (10 mmol/L), leucocytes (1100–4000 µmol/L), pituitary gland (2800 µmol/L), adrenal glands (2300 µmol/L), liver (900 µmol/L), and skeletal muscle (300–400 µmol/L) [30,31,32]. This is mainly achieved by SVCT2 active transport of ascorbate or GLUT-facilitated uptake of DHA and intracellular DHA recycling, with SVCT1 being the main co-transporter for ascorbate in the liver [30,31,32]. SVCT1 is expressed on the apical side of epithelial cells in the intestinal tract and renal tubules, being a low-affinity transporter with a *K*_m_ ≈ 65–237 µmol/L. This allows high-capacity uptake from the luminal surface of the epithelium, with a *V*_max_ of approximately 15 pmol/min/cell. In contrast, in most other cells, SVCT2 is expressed on the basolateral membrane, allowing cellular uptake from the extracellular fluid, with a high affinity (*K*_m_ ≈ 8–69 μmol/L) but low capacity (1 pmol/min/cell). SVCT2 is the primary ascorbate transporter in the brain, enabling very high tissue concentrations in neurons, even during vitamin C deficiency [30,31,32]. In mammalian vitamin C *auxotrophs*, erythrocytes lack both SVCT1 and SVCT2 and rely solely on GLUT-1 to transport DHA into the cytoplasm, where it is rapidly recycled to ascorbate by glutathione (GSH) and glutathione S-transferases (GSTs) [34]. Without rapid regeneration, DHA is irreversibly catabolized by hydrolysis to 2,3-diketogulonic acid and then to oxalate and other metabolites, including L-xylonate, L-lyxonate, or L-erythrulose [10,34]. This may explain why vitamin C *non-auxotrophic* animals with genetic deletions of L-gulono-γ-lactone oxidase (GLO) may demonstrate more severe phenotypical responses to diet-induced vitamin C deficiency, as they lack GLUT-1 facilitated erythrocyte DHA recycling. These animals also lack the additional antioxidant effects of plasma uric acid found in apes and humans. However, the loss of hepatic GLO enzyme and uricase activity developed separately during the course of higher primate evolution [35,36,37].

Administering oral liposomal vitamin C has been shown to improve serum vitamin C levels [38]. Vitamin C liposomes are nano-sized vesicles prepared from phospholipids and cholesterol, with the water-soluble vitamin C encapsulated in the aqueous core by the hydrophilic portions of the phospholipids. This has good encapsulation efficiency and allows liposome endocytosis or membrane fusion in the small intestinal mucosa, rather than the usual saturable carrier-mediated transport [39]. In an RCT which compared nanoliposomal or standard aqueous forms of oral vitamin C supplementation in healthy adult humans, oral nanoliposomal vitamin C had 1.77 times greater bioavailability, with higher values of Cmax (297.0 vs. 123.2 µmol/L), AUC 0-t (55.86 vs. 31.53 mg.h/dL), and AUC 0-∞ (78.90 vs. 57.12 mg.h/dL) [40]. However, despite this improved bioavailability, 4 g of nanoliposomal vitamin C had similar effects on the protection from ischemia-reperfusion injury when compared to 4 g of standard aqueous oral or IV vitamin C in humans with obesity [41]. The elevation of lipid peroxidation after ischemia and reperfusion, as measured by plasma concentrations of MDA, was prevented in all three modes of vitamin C delivery compared to those individuals administered placebo preparations. All participants had obesity (mean BMI = 34.1 ± 1.0 kg/m^2^) with mean baseline plasma vitamin C concentrations of 47.7 µmol/L and mean baseline thiobarbituric reactive substances (TBARS) of 5.8 ± 0.8 (µmol/L MDA) [41]. There is minimal existing literature on the effects of oral liposomal vitamin C administration on obesity or metabolic syndrome.

## 4. Vitamin C Deficiency

A low plasma ascorbate level is related to insufficient dietary intake, an accelerated rate of oxidation to DHA/catabolism, or increased redistribution or cellular uptake after trauma, surgery, or sepsis [42]. A decline in fruit juice and whole vegetable consumption in adults in the past 20 years has led to a 23% decrease in daily vitamin C intake in the USA [43]. Vitamin C deficiency, defined as a plasma ascorbic acid level of <11.4 µmol/L, was found in 7.1% of a representative sample of the USA population aged >6 years of age. Some studies define a marginal vitamin C status as plasma concentrations of 11–40 µmol/L and others as ≥11–28 µmol/L. A plasma threshold of 23 µmol/L is most commonly used to define vitamin C hypovitaminosis [42], below which preclinical symptoms and signs of scurvy (low mood, fatigue, and lethargy) may occur [6]. The EPIC-Norfolk population-based study of 22,474 UK residents reported 315 (1.4%) participants with a plasma vitamin C < 11 µmol/L and 2410 (10.7%) participants with a plasma vitamin C ≥ 11–28 µmol/L [44]. A recent analysis of the EPIC-Norfolk and the US NHANES 2017/2018 cohorts suggested that 33% of the UK EPIC-Norfolk population would not achieve an adequate plasma vitamin C concentration (50 µmol/L) by consuming the UK NHS vitamin C RDA ± 10% (i.e., 40 ± 4 mg), and 50% of the US population if they consumed the USA RDA ± 10% (i.e., 75 ± 7.5 mg for women and 90 ± 9 mg of vitamin C for men) [45].

Independent risk factors for vitamin C deficiency include older age, male gender, ethnicity (South Asians and non-Hispanic black people), lower physical activity, institutionalized or isolated people, socioeconomic disadvantage, inadequate diet, and lower education level [43,44,46]. SVCT-1 and SVCT-2 expression decreases with age, which impairs the absorption and distribution of vitamin C in the body in older persons [47,48,49]. Loss of ascorbate in the urine is increased in patients with T2DM [17]. Smokers and individuals with obesity, T2DM, or hypertension are more likely to have vitamin C deficiency due to greater systemic inflammation, ROS generation, and oxidative stress. This consumes vitamin C, as it is an antioxidant required for free radical scavenging and the regeneration of other antioxidants, including glutathione, α-tocopherol (vitamin E), urate, and β-carotene [19,50,51]. Lipid peroxidation and advanced lipoxidation end-products (ALEs), and protein oxidation and carbonyl stress with advanced glycation end-products (AGEs) formation increase with age, obesity, and unhealthy diets [52]. Carbonyl and oxidative stress contribute to vitamin C depletion and age-related comorbidity. This is reflected in the French (AFSSA) nutritional guidelines having a higher recommended vitamin C intake for adults aged 75 years and older (120 mg/d), which considered the higher risks of cancer, cardiovascular disease, cognitive impairment, and declining immunity with age [23,53].

Vitamin C deficiency is prevalent in hospital intensive or acute care patients, medical oncology and geriatric inpatients, or after major surgery [54]. A meta-analysis showed the pooled cumulative prevalence of vitamin C deficiency amongst hospital inpatients from high-income countries was 28% (n = 2494; 95% CI, 21–34). The prevalence of scurvy signs in the inpatients with vitamin C deficiency was reported in two studies and ranged from 48% (n = 29) to 62% (n = 42) [54]. In a study of 309 Australian outpatients presenting preoperatively to a surgical service, 21% were found to have vitamin C deficiency (<11.4 µmol/L), and 43% of the cohort had plasma concentrations below 28.4 µmol/L. In the overall cohort, 11% of patients ate no fruit, with lack of fruit consumption being an independent risk factor for plasma vitamin C < 28.4 µmol/L on multivariate analysis [44]. Type 2 diabetes mellitus is also a significant independent predictor of vitamin C deficiency due in part to higher levels of oxidative stress, obesity, metaflammation, poor diet, and renal leak of urinary vitamin C [17,55]. The renal leak of vitamin C occurred in 9% of non-diabetic controls compared with 33% of participants with T2DM (OR: 5.07; 95% CI: 1.97, 14.83; *p* < 0.001), with a significantly lower mean plasma vitamin C concentration in participants with T2DM (53.1 µmol/L control vs. 40.9 µmol/L T2DM, *p* < 0.001). Risk factors for vitamin C renal leak included elevated fasting plasma glucose, glycosylated hemoglobin A1c, BMI, micro/macrovascular complications, and protein/creatinine ratio [17].

## 5. Vitamin C and Obesity

Obesity can substantially affect the level of vitamin C in the body [56,57,58,59]. Individuals with a body weight above 91 kg have significantly lower plasma vitamin C concentrations compared to those less than 72 kg in weight [24]. Plasma vitamin C levels have been reported to be inversely associated with BMI and waist circumference in both adult men and women [56,58]. This association was also reported in children and adolescents. For example, a high prevalence (44%) of vitamin C deficiency was reported in children with obesity in Mexico, with the vitamin C level found to be inversely associated with BMI, waist-to-height ratio, and percentage of body fat [57]. In a study of 266 patients undergoing abdominal surgeries, including bariatric surgery, preoperative vitamin C deficiency (<17 µmol/L) was found in 15% of patients and depletion (<34 µmol/L) in 17% of patients. The mean BMI was 39 ± 11 kg/m^2^, with vitamin C concentrations being inversely associated with increasing BMI (*p* = 0.02). Importantly, this association was independent of dietary supplementation, ascorbic acid supplementation, or daily vegetable/fruit intake (*p* = 0.01) [60].

In the 2005 EPIC study of 19,068 men and women without a known chronic illness, the inverse relationship between plasma vitamin C levels and the abdominal distribution of adipose tissue (waist-to-hip ratio, WHR) was independent of other risk factors, including BMI, age, vitamin supplement use, cigarette smoking, and socioeconomic status [56,61]. A 2023 meta-analysis of observational studies suggested a higher dietary intake of vitamin C was significantly associated with lower BMI (MD = −0.5 kg/m^2^, 95% CI: −0.2, −0.9; I^2^ = 97%) but was not significantly associated with WC (n = 1 study only, MD = −3.4 cm, 95% CI: −7.4, +0.6). In comparison, high plasma ascorbate concentrations were more strongly associated with a lower BMI (MD = −1.4 kg/m^2^, 95% CI: −0.7, −1.4; I^2^ = 86%) and WC (MD = −4.5 cm, 95% CI: −2.9, −6.1, I^2^ = 90%) [62].

**Figure 3:**
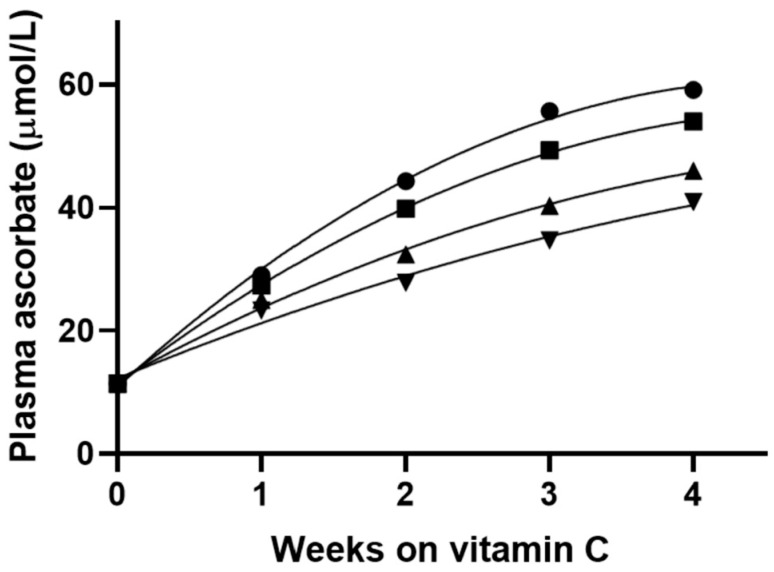
Predicted plasma ascorbate concentration after repletion with vitamin C (117 mg/day) by body weight in non-smoking male subjects. The body weights used in the model were: 59 kg (130 lbs; ●), 68 kg (150 lbs; ■), 82 kg (180 lbs; ▲), 91 kg (200 lbs; ▼). The model assumed that all participants started at the same depleted ascorbate plasma level of 12 µmol/L. Even after 4 weeks of supplementation with 117 mg/day of vitamin C, patients with body weights above 80 kg did not reach sufficient steady-state plasma ascorbate concentrations for optimal tissue saturation after previous depletion–repletion cycles. Reproduced from Carr et al. (2022) with permission [22,61].

Extra vitamin C is required for patients with obesity to achieve adequate plasma vitamin C concentrations of 50 µmol/L. An additional 10 mg/day of vitamin C supplementation is required for every 10 kg increase in weight to achieve the equivalent plasma vitamin C concentration (50 µmol/L) in a 60 kg individual who meets the current EFSA RDA of 110 mg dietary vitamin C intake [22,61]. Even after 4 weeks of supplementation with 117 mg/day of vitamin C, individuals with body weights above 80 kg do not achieve sufficient steady-state plasma ascorbate concentrations for optimal tissue saturation after previous vitamin C depletion–repletion cycles (Figure 3) [22,61]. In a subsequent analysis of US NHANES 2017/2018 data involving 2828 participants, an additional 24 mg/day and 22 mg/day of vitamin C per extra 10 kg gained is respectively required for smokers and non-smokers with obesity (>100 kg) to be vitamin C sufficient. This is believed to be the result of the underlying volumetric dilution, enhanced inflammation, and oxidative stress associated with obesity and has important implications for global vitamin C dietary recommendations, clinical research, and patient care [24].

## 6. Vitamin C Supplementation in Obesity

Randomized trials assessing the effect of vitamin C supplementation in patients with obesity have found modest or no overall improvements in weight or features of metabolic syndrome [63,64,65,66,67]. This is despite large observational studies showing that plasma vitamin C concentrations are inversely associated with BMI and waist circumference [56,58], as well as the EPIC-Norfolk 12-year prospective prevention study reporting a significantly lower risk of new-onset T2DM in individuals with higher plasma concentrations of ascorbic acid (odds ratio: 0.38; 95% CI: 0.28, 0.52) [68].

The Women’s Antioxidant Cardiovascular Study (1995–2005) was an RCT of micronutrient supplementation in 6574 women at high risk of cardiovascular disease, of whom 73.3% were either overweight or obese [69]. At a median follow-up of 9.2 years, there was no significant overall reduction in incident T2DM with 500 mg/day of vitamin C supplementation (RR: 0.89, 95% CI: 0.78, 1.02; *p* = 0.09). However, there was a significant reduction in future T2DM risk in women with no hypercholesterolemia history (RR: 0.64, 95% CI: 0.48, 0.87). In addition, the overall cumulative incidence of T2DM began to separate after 5 years of supplementation with vitamin C compared to placebo but did not reach statistical significance (*p* for log-rank test = 0.09) [69].

A 2017 metanalysis of 22 RCTs of oral vitamin C supplementation in glycemic control did not find any overall improvement in glucose, HbA1c, and insulin concentrations [64]. However, on subgroup analysis, vitamin C supplementation had significant effects on fasting plasma insulin concentrations (−13.63 pmol/L, 95% CI: −22.73, −4.54, *p* < 0.01) but not on postprandial insulin concentrations. A longer vitamin C intervention (>30 days) had a greater effect on fasting glucose (−0.53 mmol/L, 95% CI: −0.79, −0.10, *p* = 0.02). In patients with T2DM, patients in the vitamin C intervention group had reduced glucose concentrations (−0.44 mmol/L, 95% CI: −0.81, −0.07, *p* = 0.01). Meta-regression analysis showed patients with obesity/overweight, older patients, and those with higher baseline plasma glucose had greater responses to vitamin C administration [64]. Observational studies are limited by the poor reliability of nutritional surveys and patient recall and by their cross-sectional nature [62]. The outcomes of vitamin C supplementation studies can be affected by vitamin C dosage and length of administration, as well as the baseline nutritional status and plasma vitamin C levels of participants. For example, 6 years of multivitamin supplementation reduced cerebrovascular mortality in a population with low baseline micronutrients, and vitamin C supplementation improved the vascular health of patients with low consumption of fruit and vegetables [64]. A placebo-controlled RCT of 2 months of supplementation with daily 500 mg oral vitamin C or vitamin C, α-lipoic acid, and 550 IU α-tocopherol (vitamin E) was conducted in smokers [70]. The lipid peroxidation biomarker F_2_-isoprostane was significantly reduced by antioxidants in smokers with a high BMI (>26.6 kg/m^2^) but not in smokers with a normal BMI. Although this reduction in F_2_-isoprostane was numerically greater with the combined antioxidants (−36.5 pmol/L) than with vitamin C monotherapy (−28.8 pmol/L), the effect was not significantly different between the two supplements [70]. It has been suggested that vitamin C deficiency may be a surrogate marker for poor diet and unhealthy lifestyle rather than a true causative factor in obesity [71].

Combining vitamin C with other vitamins and minerals to improve the micronutrient profile and antioxidant capacity of patients with obesity has also been trialed [64,65,66]. Based on previous findings that higher-risk patients may have greater benefits with vitamin C supplementation [72], Vlasiuk et al. conducted a double-blind, placebo-controlled RCT comparing a daily oral micronutrient tablet supplement to a closely matched (for appearance and flavor) placebo tablet [66]. This involved 72 participants who were predominantly non-smoking females with a median BMI = 39 kg/m^2^ (Q1, Q3, 34, 42) and features of metabolic syndrome [66]. The micronutrient supplement contained 1000 mg of vitamin C, 10 ug of vitamin D, 45 mg of vitamin E, 700 ug of vitamin A, 6.5 mg of vitamin B6, 400 ug of folate, 9.6 ug of vitamin B12, 10 mg of zinc, 5 mg of iron, 0.9 mg of copper and 110 ug of selenium. At baseline, the median plasma vitamin C (Q1, Q3) concentration was 29 (15, 41) µmol/L. Only 10% of patients had adequate baseline plasma vitamin C concentrations (i.e., ≥50 µmol/L), 90% had inadequate concentrations (<50 µmol/L), 38% had hypovitaminosis C (≤23 µmol/L), and 19% had outright deficiency (≤11 µmol/L). The participants had been pre-screened to have a CRP of ≥3 mg/L, which is associated with a higher risk of cardiovascular disease. The study found that 12 weeks of micronutrient supplementation was unable to significantly improve the elevated baseline median concentrations of the inflammatory biomarker CRP (5.6 (3.6, 8.8) mg/L) or the elevated inflammatory cytokines IL-6 (9.9 (6.7, 15) pg/mL) or TNFα (0.48 (0, 3.3) pg/mL). The median baseline level of F_2_-isoprostane (1.4 (0.4, 2.3) ng/mg creatinine) was in the normal range and unchanged by vitamin C supplementation. Importantly, 30% of patients in the intervention group did not achieve adequate plasma levels of vitamin C (>50 µmol/L) even after 12 weeks of micronutrient supplementation. Micronutrient supplementation resulted in small but statistically significant improvements in glycemic control (fasting glucose (Δ−0.3 [95%CI −0.6, −0.05] mmol/L, *p* = 0.02), HbA1c concentration (Δ−2.1 [95%CI −3.6, −0.6] mmol/L, *p* = 0.007), and Homeostatic Model Assessment of insulin sensitivity (HOMA%S) (Δ12 [95%CI 0.1, 23]%; *p* = 0.049), with a trend to lower fasting insulin concentrations (Δ−60 [95%CI −122, 1.7] pmol/L, *p* = 0.06). The micronutrient intervention group experienced a stabilization of BMI and body weight, with the control group continuing to gain weight (Δ0.9 [95%CI −0.2, 1.9] kg, *p* = 0.1) and BMI (Δ0.4 [95%CI 0.0, 0.8] kg/m^2^, *p* = 0.04). This was associated with an increased metabolic syndrome severity (MetS) z score in the control group of 0.2 (95%CI 0.02, 0.40), *p* = 0.03) based on BMI, and 0.2 (95%CI 0.02, 0.3), *p* = 0.03) based on WC [66]. Micronutrient supplementation for 12 weeks improved some metabolic indices but was insufficient to control oxidative stress, insulin resistance, hyperlipidemia, and metabolic syndrome in obesity, and the addition of dietary and weight loss interventions was required [66].

## 7. Vitamin C and NAFLD

The worldwide burden of chronic liver disease due to NAFLD is in parallel with the increased prevalence of obesity, metabolic syndrome, and T2DM [73,74]. NAFLD has recently been renamed metabolic dysfunction-associated steatotic liver disease (MASLD) and includes the spectrum of hepatic steatosis, steatohepatitis (MASH), fibrosis, cirrhosis, and hepatocellular carcinoma [75]. Exposure to lipotoxic palmitate and the intracellular accumulation of triglycerides causes stress-induced apoptosis and senescence in hepatocytes. This stimulates the release of exosomes and chemokines, hepatic stellate cell activation, collagen synthesis, and fibrogenesis [76]. A reduction of 5% in BMI resulted in a 25% decrease in liver fat as estimated by MRI proton density fat fraction [77]. In a year-long study of calorie restriction in 261 patients with biopsy-proven non-alcoholic steatohepatitis (NASH), a loss of ≥5% body weight resulted in a greater reduction in the NAFLD activity score (NAS) compared to a weight loss of <5% [77]. Those who lost ≥10% body weight all showed reductions in the NAS; 90% had NASH resolution, and 45% showed fibrosis regression. This degree of sustained weight loss may not be attainable in the majority of patients without the addition of pharmacological interventions or bariatric surgery to conventional lifestyle changes (dietary intervention and >150 min exercise/wk) [77].

Vitamin C is important in maintaining normal adiponectin secretion, cholesterol transport and excretion, energy metabolism, and the reduction of oxidative stress. All of these are deranged in NAFLD, with hepatic insulin resistance, lipid peroxidation, de novo lipogenesis, hepatocyte oxidative stress and lipid accumulation, macrophage and neutrophil infiltration of the liver, and activation of hepatic stellate cells being involved in the development of NASH and the progression to fibrosis. Animal studies using vitamin C for the prevention and/or treatment of NAFLD have shown dramatic results. For example, in high-fat diet (HFD, 45% kcal fat) fed wild type C57BL/6 male mice, oral vitamin C supplementation (1% w/w) for 15 weeks significantly increased mRNA levels in visceral adipose tissue (VAT) of peroxisome proliferator-activated receptor alpha (PPARα) by 49%, acyl-coenzyme A oxidase (ACOX) by 60%, carnitine palmitoyltransferase I (CPT-1) by 44%, very long-chain acyl-CoA dehydrogenase (VLCAD) by 35%, and significantly increased respective hepatic PPARα, ACOX, CPT-1, and VLCAD mRNA levels by 17%, 22%, 45% and 12% [78]. Compared to HFD-fed control animals, vitamin C supplementation increased serum vitamin C concentrations by 41% which;

- Promoted visceral adipocyte and hepatocyte fatty acid oxidation;
- Improved visceral adipocyte hypertrophy (by 24%) and VAT mass (by 21%);
- Decreased hepatocyte triglyceride content (79% decrease in histological hepatic steatosis) to that of LFD (10% kcal fat)-fed mice;
- Reduced markers of NASH and hepatocyte injury (decreased ALT and AST levels by 35% and 36%);
- Reduced serum concentrations of total and LDL cholesterol by 24% and 50%, respectively;
- Inhibited hepatic cytokine release of tumor necrosis factor α (TNF-α) and MCP-1 by 36% and 34%, respectively;
- Decreased hepatocyte apoptotic caspase 8 mRNA levels by 73% and increased anti-apoptotic B-cell lymphoma 2 (Bcl-2) mRNA levels by 84%;
- Decreased the expression of genes involved in hepatic fibrosis, transforming growth factor β (TGF-β) and collagen α1 by 26% and 29%, respectively;
- Prevented HFD-related body weight gain by 26% [78].

Other animal models aimed to replicate human vitamin C auxotrophy using animals with inherited germline or engineered mutations in vitamin C synthesis. In HFD (20% fat, 15% sucrose, 0.35% cholesterol)-induced obesity in guinea pigs, vitamin C administration (2000 mg of vitamin C/kg feed) was unable to prevent weight gain, the progression of NASH, or hyperlipidemia (triglycerides, total cholesterol) at 16 weeks. However, after changing to a low-fat diet, vitamin C deficiency was associated with the delayed regression of NASH as measured by liver histology (steatosis, inflammation, and hepatocyte ballooning) (*p* < 0.05 or less) and NASH transcriptome [79].

Vitamin C therapy has not been as successful in RCTs involving humans with obesity and NAFLD compared to murine models, with vitamin C monotherapy being unable to improve hyperlipidemia, cytokine release, hepatic steatosis, cholesterol excretion, FAO, and obesity to the same extent. In an RCT involving 84 patients with obesity and NAFLD, oral vitamin C supplementation with 250 mg, 1000 mg, or 2000 mg/day for 12 weeks was compared. This was higher than the Chinese Residents’ Dietary Guidelines proposed vitamin C intake for chronic non-communicable diseases (PI-NCD) of 200 mg/day. The medium dose of 1000 mg/day was able to improve markers of hepatocyte injury (AST −5.00 (−10.25, −1.75) U/L, *p* < 0.001, ALT −8.00 (−18.00, −1.75) U/L, *p* < 0.05), the homeostasis model assessment for insulin resistance (HOMA-IR, baseline 4.61 ± 3.51 vs. after 2.62 ± 2.41, *p* < 0.001), total serum adiponectin (baseline 30.57 ± 17.93 µg/mL vs. after 78.48 ± 50.24 µg/mL, *p* < 0.001), and HMW adiponectin (baseline 4.18 ± 2.54 µg/mL vs. after 9.09 ± 4.31 µg/mL, *p* < 0.001), although significant improvements in total and HMW adiponectin occurred with all three dosages of vitamin C. It was postulated that this vitamin C-related elevation of HMW adiponectin could reduce TNF-α related hepatocyte stress by activation of the FGF21/FGFR2/adiponectin pathway, as demonstrated in previous in vitro models. There was no significant improvement in total serum cholesterol or triglycerides. No liver biopsies or imaging to confirm histological or ultrastructural changes were performed, and there was no placebo control group. The authors concluded that 1000 mg/day of vitamin C was the optimal dose to improve markers of liver function and metabolic health in patients with NAFLD [80].

When comparing the effects of vitamin C in different studies, some potential confounders include interspecies differences in lipid handling, bile salt synthesis, cholesterol excretion, and FAO, the inability to achieve adequate vitamin C serum levels during supplementation, or the lack of significant weight loss in humans to augment metabolic improvements. In addition, despite the association between human NAFLD and serum vitamin C concentrations in large population studies, causation has not been proven [81,82]. This is reflected in the substantial improvement in NASH after treatment with metabolic bariatric surgery, GLP-1R agonists (semaglutide), glucagon receptor/GLP-1R dual agonists (survodutide), or specific hepatic thyroid hormone receptor β agonists (resmetirom) [83,84,85,86]. Several other clinical RCTs using pharmaceutical monotherapy for NASH and NASH fibrosis have failed to achieve primary endpoints, including the lysyl oxidase homolog 2 (LOXL2) inhibitor simtuzumab, the apoptosis signal-regulating kinase 1 (ASK1) inhibitor selonsertib, the dual antagonist of chemokine receptors 2 and 5 cenicriviroc, or the polyethylene glycol-conjugated analog of human fibroblast growth factor 21 pegbelfermin [87].

Vitamin C intake contributes to the maintenance of glycolipid metabolism, thermoregulation, and antioxidant defenses and may be synergistic with other treatments for NAFLD in obesity, including calorie restriction and vitamin E supplementation. Vitamin E monotherapy has not been shown to reduce NASH fibrosis in 15 clinical RCTs but may have clinically meaningful improvements in NASH in monotherapy or in combination with vitamin C, spironolactone, or pioglitazone (PPAR-γ agonist) in RCTs [88,89,90,91,92]. However, there have been some concerns raised about the long-term risks of fat-soluble vitamin E supplementation at oral doses above the recommended upper limit of 1500 IU/day, as well as the side effects of pioglitazone. The American Association for the Study of Liver Diseases 2018 guidelines suggested the use of vitamin E therapy only in patients with biopsy-proven NASH without diabetes [88,89,90,91,92]. Combined trials of vitamin C and E are limited by trial design, including small sample size, heterogeneous methodology, and lack of monotherapy comparator arms or paired liver biopsies [88,89,90,91,92].

## 8. Conclusions

Humans have lost the ability to manufacture ascorbic acid from glucose, and thus, vitamin C is an essential dietary micronutrient. Due to its reducing ability, vitamin C acts as a small molecule anti-oxidant and as a required co-factor for numerous transition metal-dependent enzyme reactions in the body. There is great variability between different nutritional guidelines in the recommended daily allowance of vitamin C. Vitamin C deficiency results from inadequate intake of fresh fruit and vegetables, and also increased utilization by oxidative and carbonyl stress. Risk factors for vitamin C deficiency include cigarette smoking, malnutrition, age, obesity, T2DM, hypertension, race, sex, social isolation, major surgery, and Western diets. Despite the common belief that vitamin C deficiency is rare in affluent countries, surveys of large populations and specific patient groups suggest otherwise. Persons with obesity typically consume highly processed, energy-dense foods which contain inadequate micronutrients. As obesity progresses, larger amounts of oral vitamin C are required to achieve adequate plasma and tissue concentrations, as compared to persons with a healthy weight. Randomized controlled trials have demonstrated that vitamin C replacement may not be sufficient to correct plasma vitamin C levels and resolve oxidative stress, adipose tissue inflammation, or features of metabolic syndrome in human obesity. Combining micronutrient supplementation and healthy whole-food diets with effective weight loss interventions may be synergistic in improving glucose tolerance, insulin resistance, dyslipidemia, NAFLD, and adiponectin secretion.
